# Supplementary material for: Digital dissection of the pelvis and hindlimb of the red-legged running frog, Phlyctimantis maculatus, using Diffusible Iodine Contrast Enhanced computed microtomography (DICE μCT)
Source: PeerJ. 2019 Jun 7;7:e7003. doi: 10.7717/peerj.7003 (PMC6557250; doi:10.7717/peerj.7003)

***Phlyctimantis maculatus* digital dissection 3D PDF user guide**

Upon opening the 3D PDF file, the following banner may appear, in which case the user will need to confirm the file is trusted.

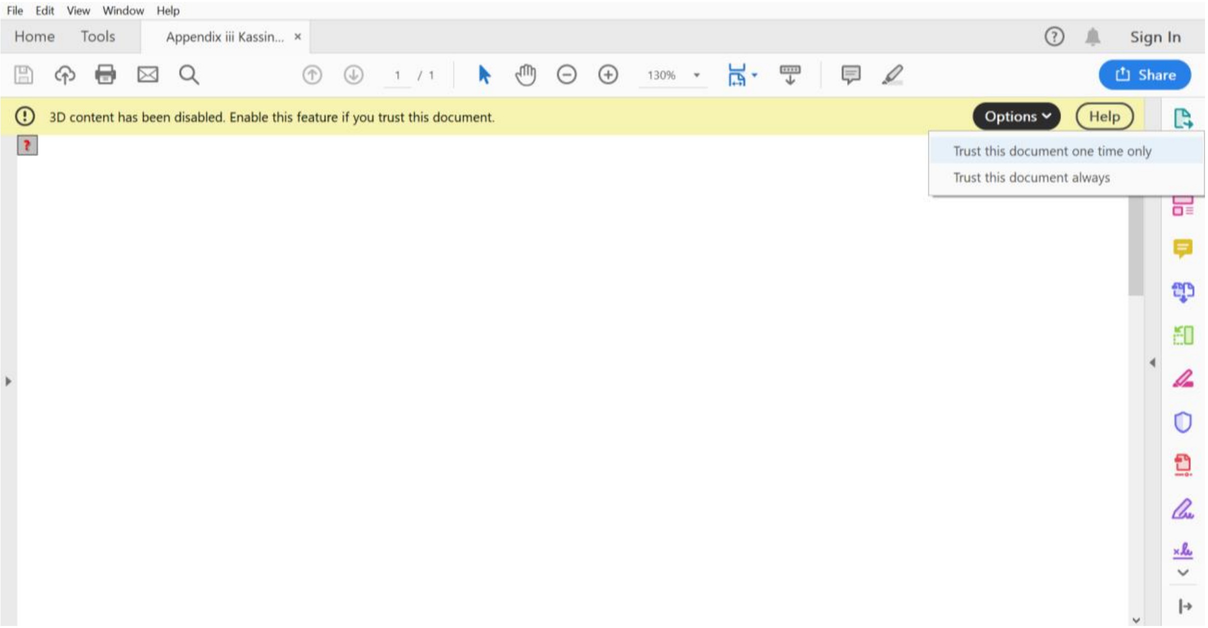

To open the model into view, click on the blank screen. The 3D model should then appear in the default view.

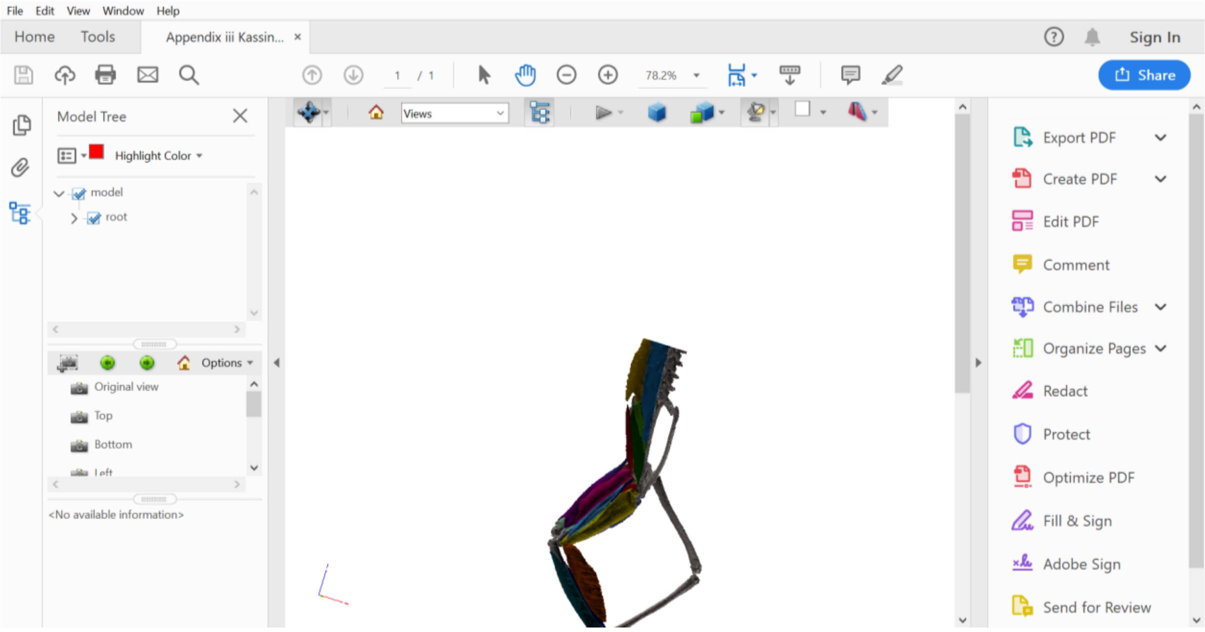

To change the lighting click on the ‘enable extra lighting’ button which appears as a small lamp icon in the tool bar and select an option, we recommend the ‘CAD Optimized Lights’ option.

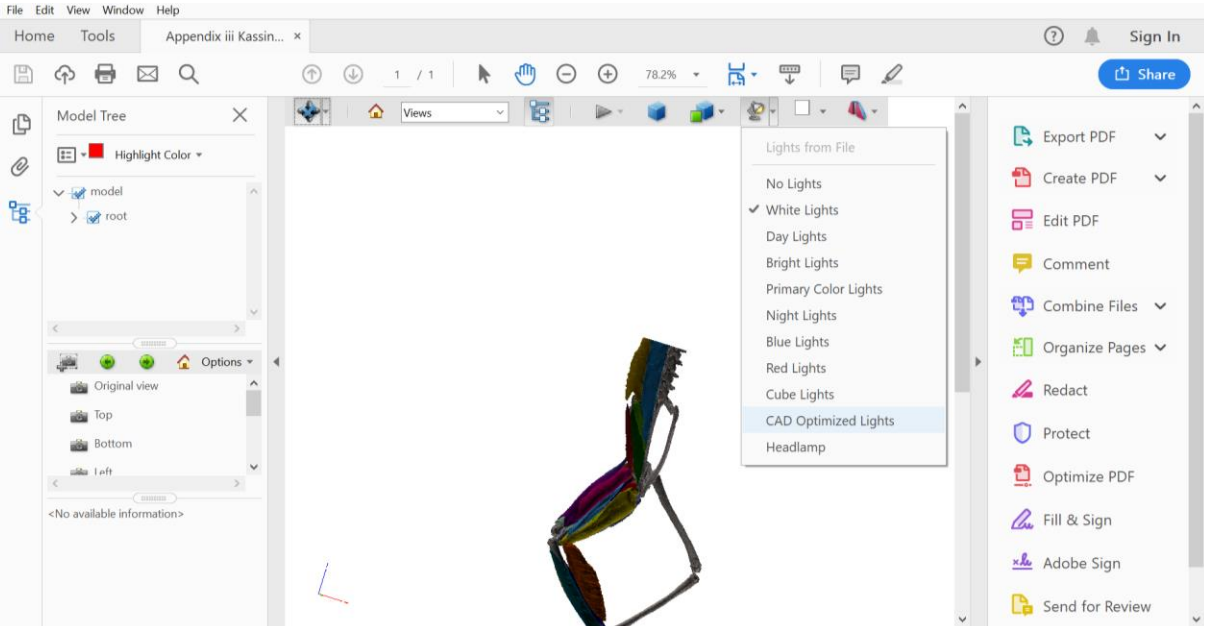

To pan and centre the model, select the pan tool from the 3D tools menu and hold left click while moving the mouse.

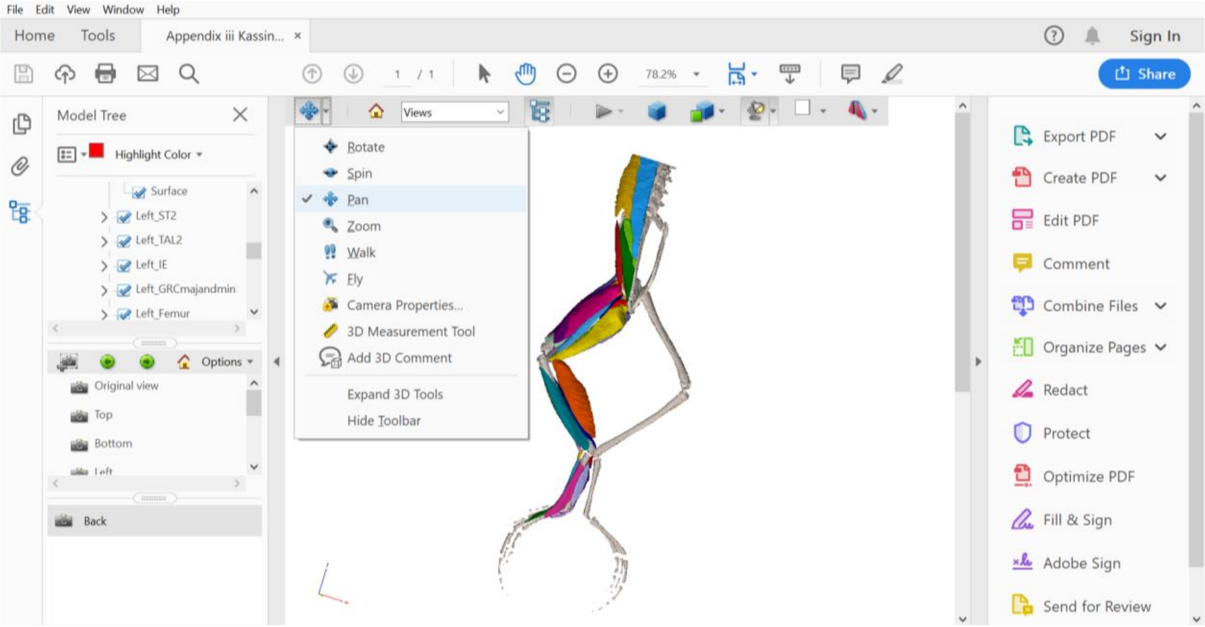

To zoom in and out, use the wheel of the mouse or hold down right click while moving the mouse up and down. Alternatively, select the zoom tool from the 3D tools menu and hold left click while moving the mouse up and down.

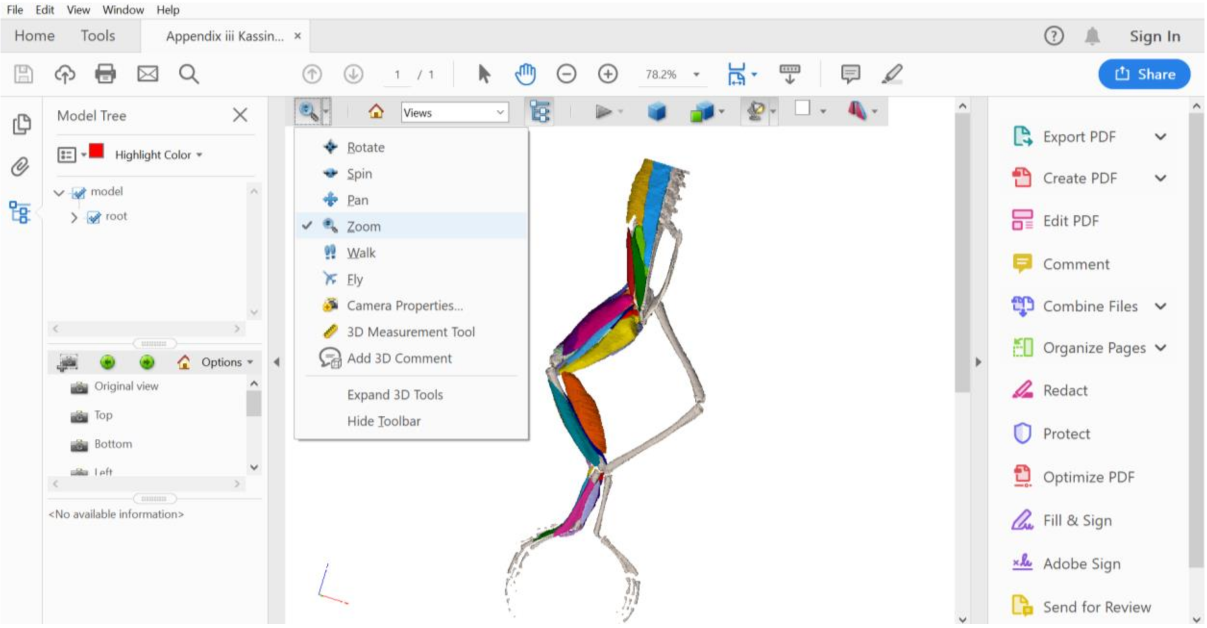

To rotate the model, hold left click and manipulate the mouse with the rotate 3D tool selected.

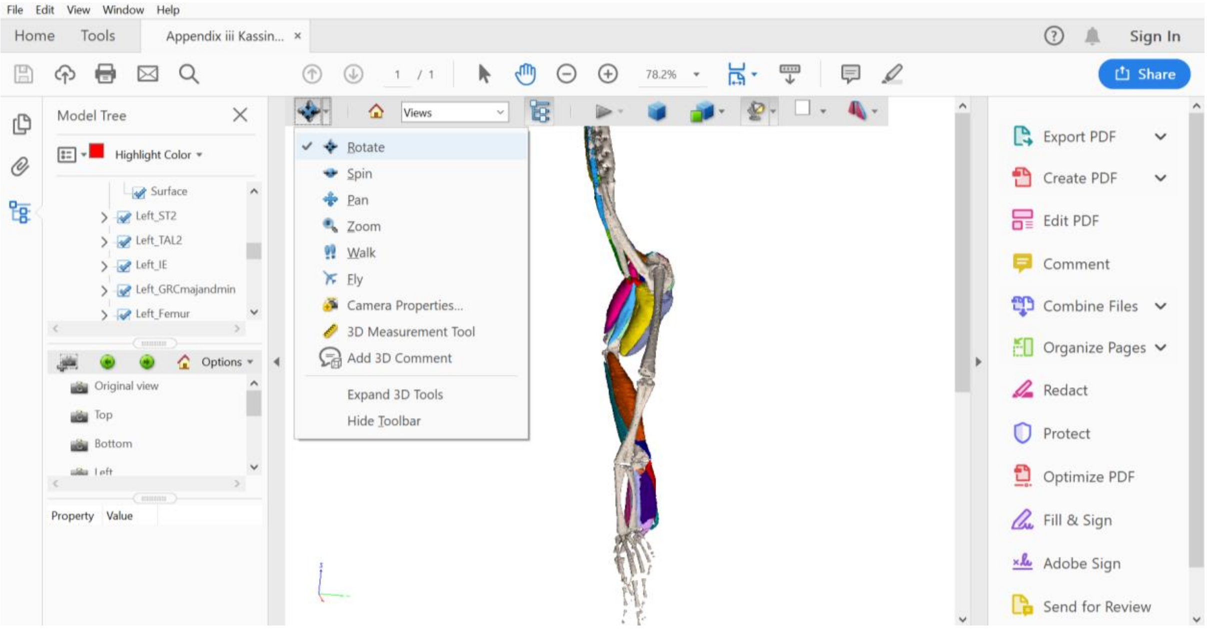

Pre-set views can also be selected using the 'Views' drop down menu. Be aware that selecting a pre-set view will reset the lighting and positioning of the model, which are easily changed as per instructions above. 'Original view' provides a dorsal view of the model; 'Top' provides a cranial-caudal transverse view of the model ventral side up; 'Bottom' provides a caudal-cranial transverse view of the model with the ventral side up; 'Left' provides a lateral view of the left side of the model; 'Right' provides a lateral view of the right side of the model; 'Front' provides a dorsal view of the model; 'Back' provides a ventral view of the model.

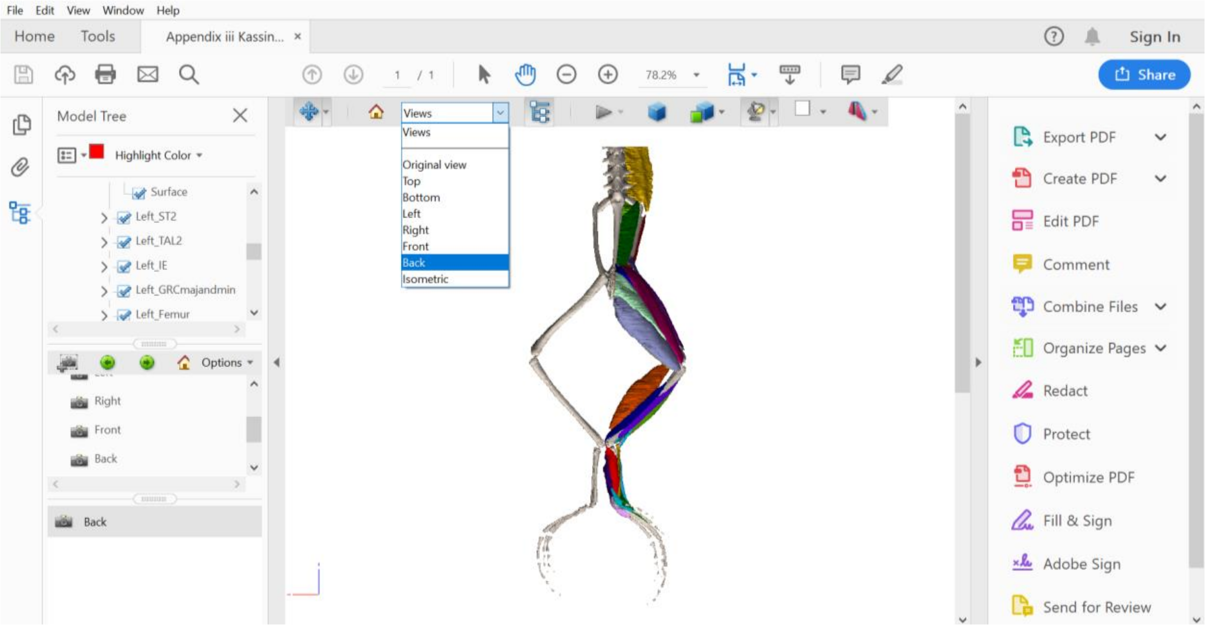

The default view can be returned to at any time by clicking the 'Default View' button appearing as a small house icon in the tool bar.

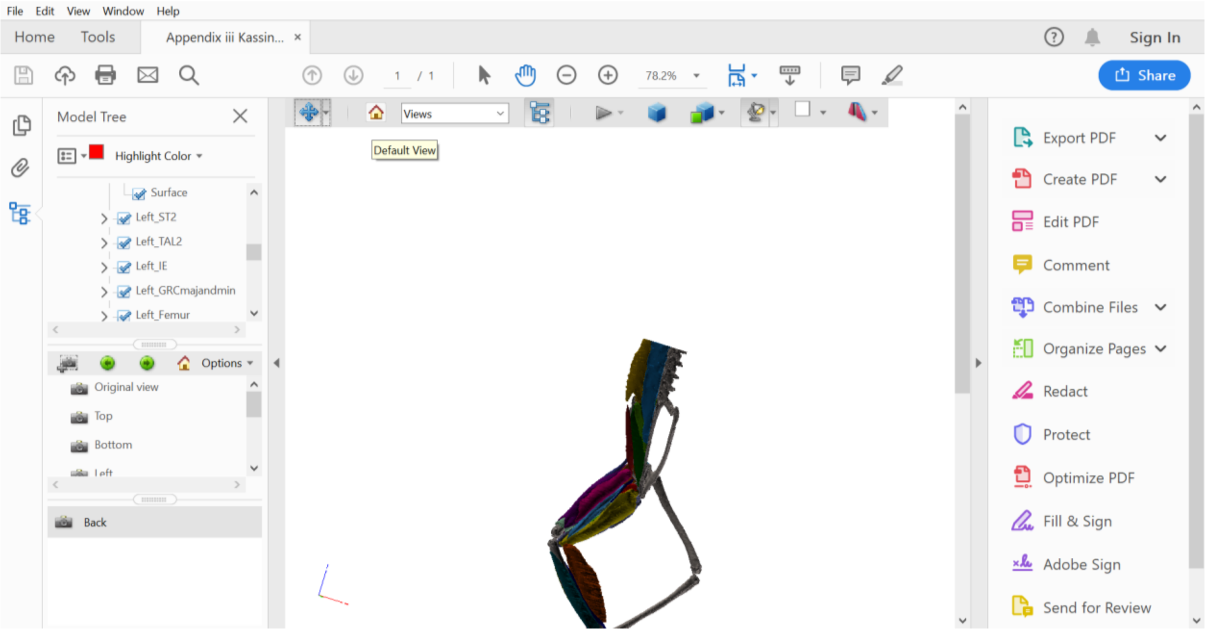

To perform a digital dissection by hiding and revealing the separate muscular and bony elements from view, the model tree needs to be displayed. The model tree may be already open, but in cases where it is not, select the ‘Toggle Model Tree’ button on the tool bar which appears as a blue branching diagram. Alternatively go to view > Show/Hide > Navigation panes and then select Model Tree.

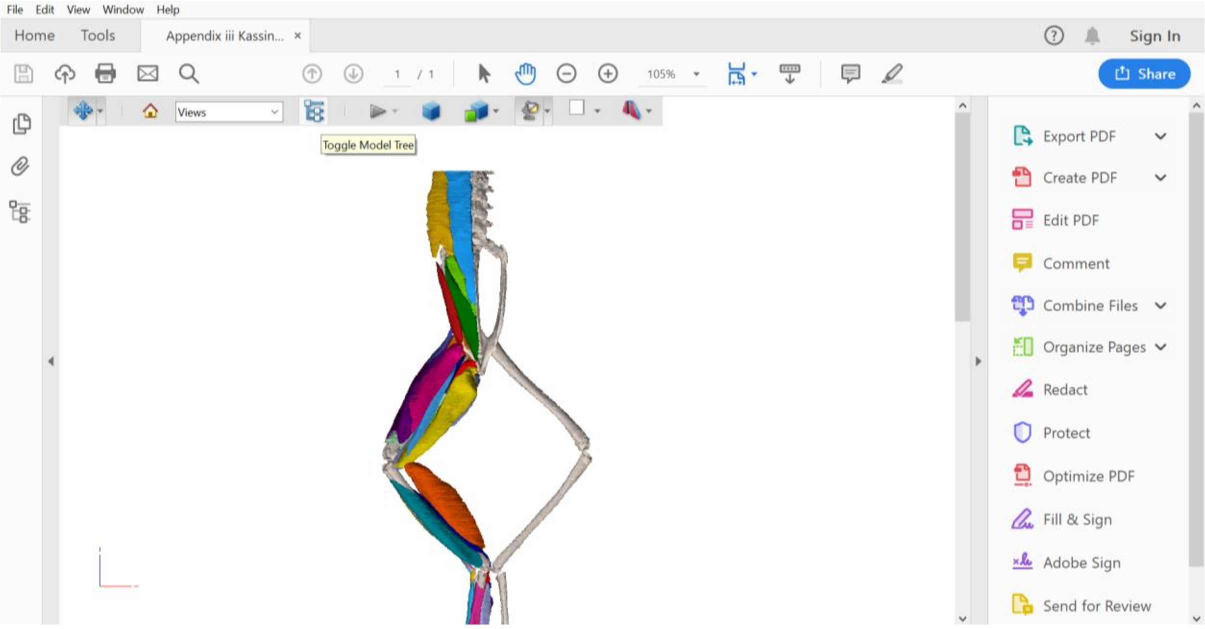

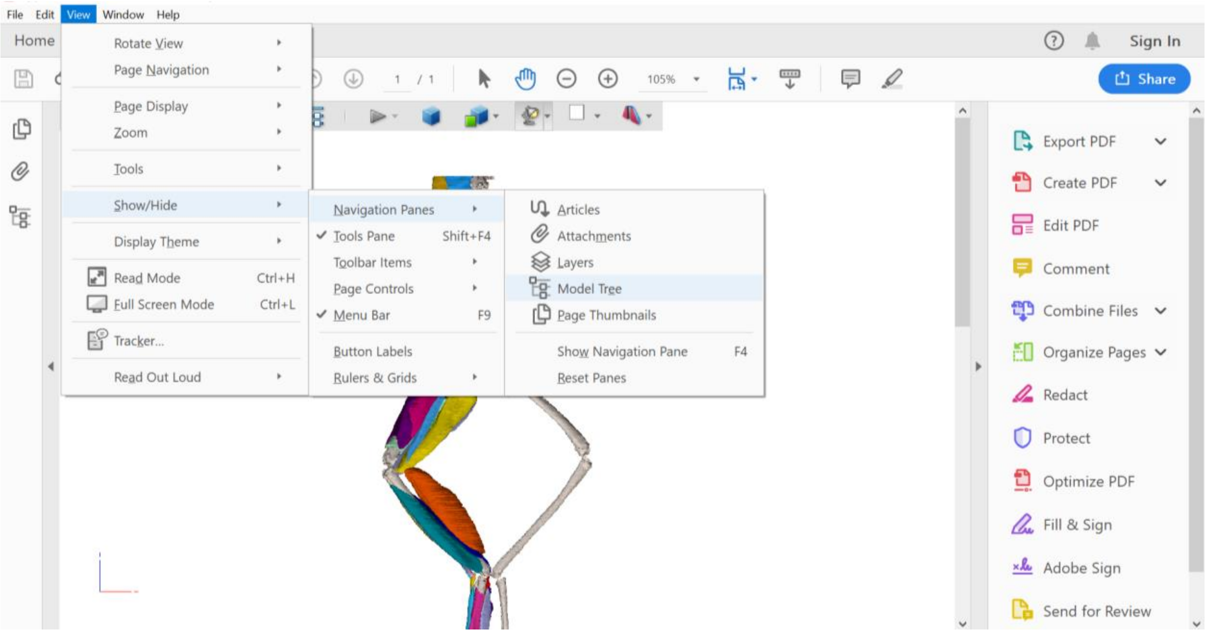

The Model Tree pane will appear on the left hand side, to view a list of all of the muscles and bones the small arrow next to the ‘root’ checkbox should be clicked to reveal the parts menu.

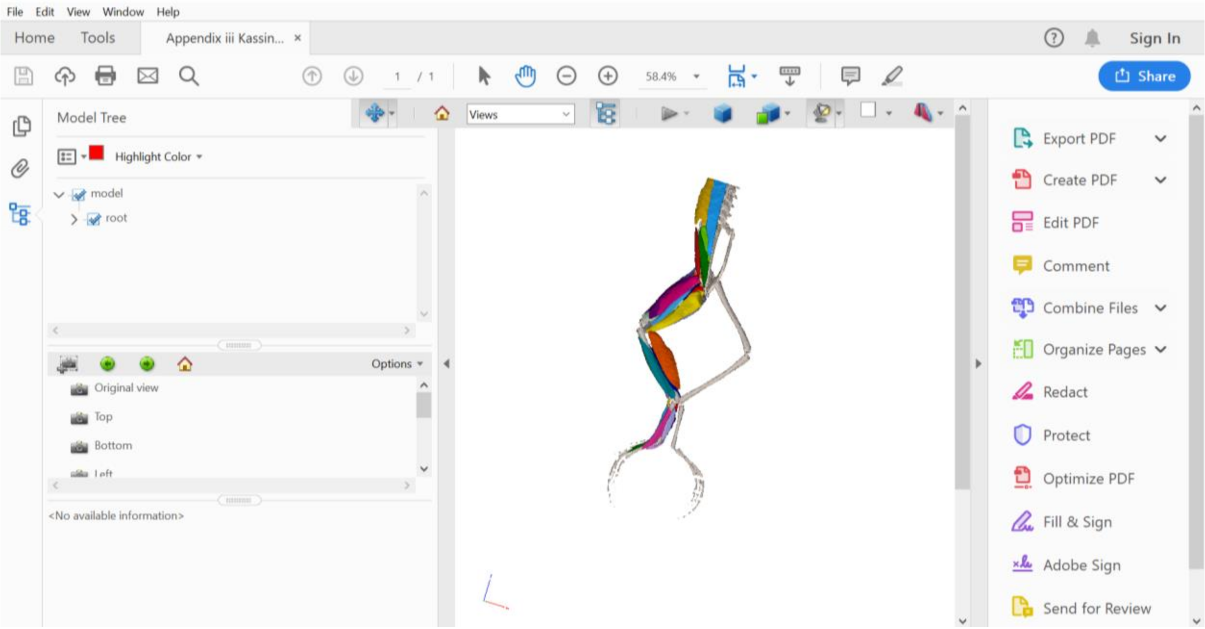

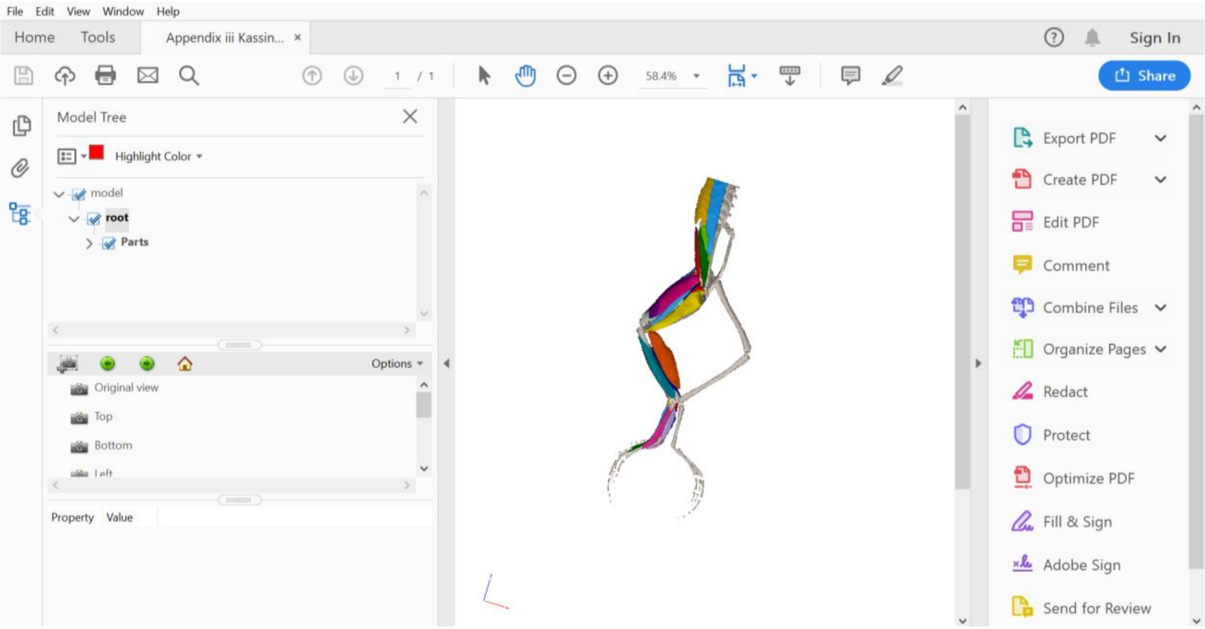

Click the small arrow next to the 'Parts' checkbox to display a list of all the muscles and bones will be listed alongside their own checkboxes.

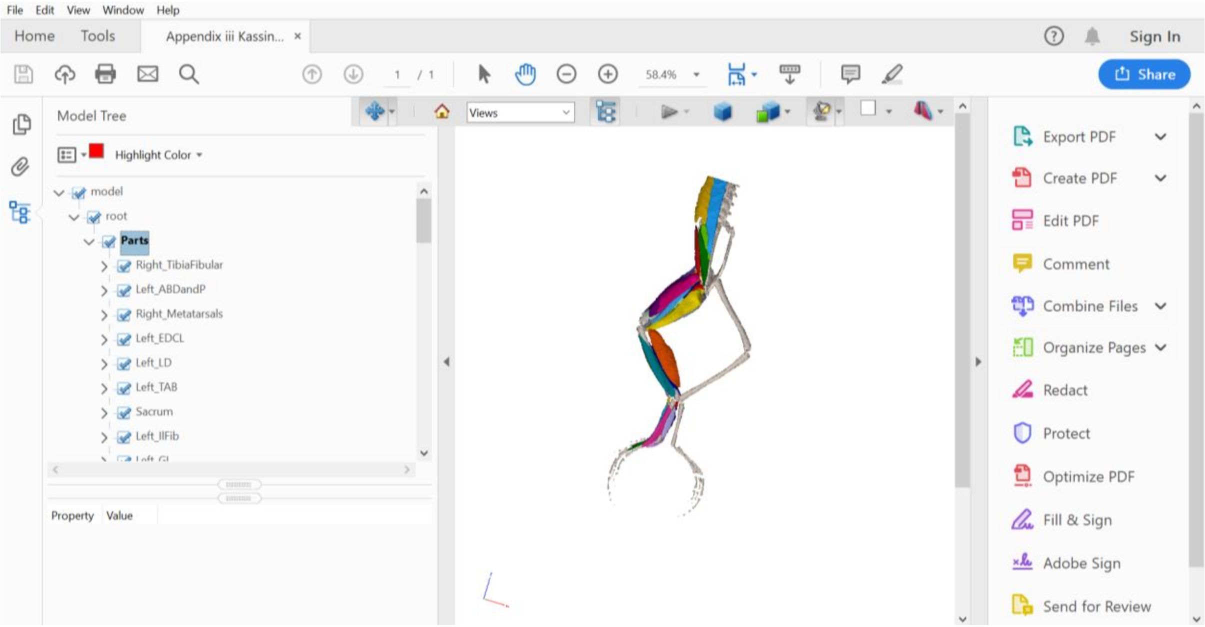

As default all elements will be checked on and visible. To hide a muscle or bone, uncheck the box next to the muscle or bone of interest.

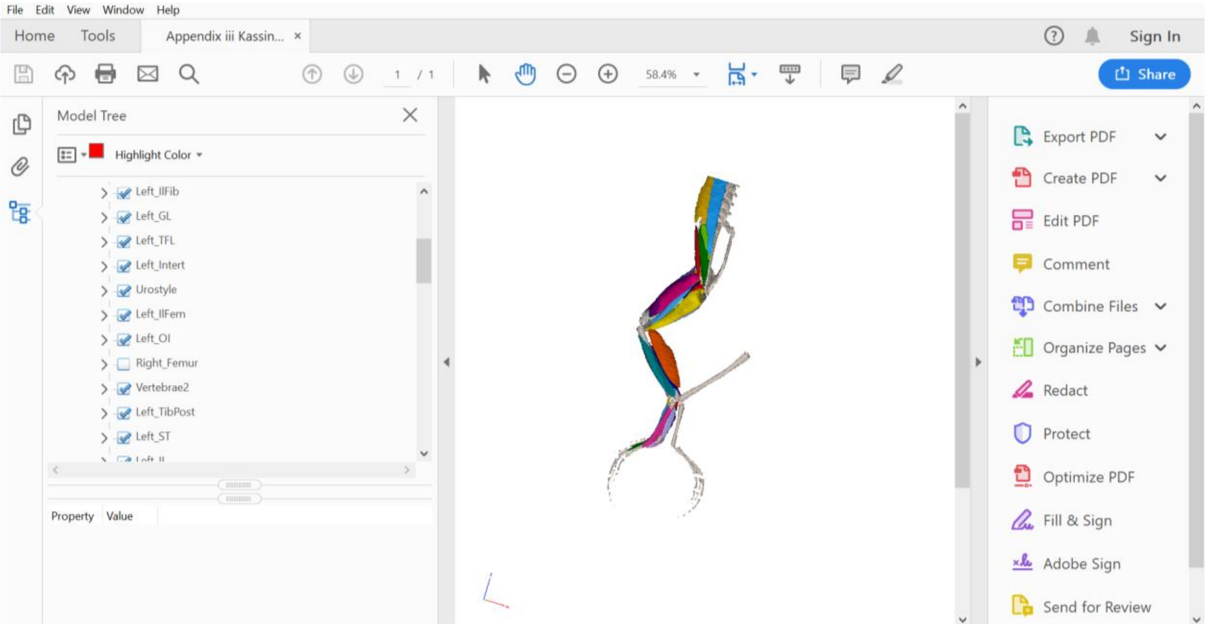

Re-checking the box will bring the muscle or bone back into view again.

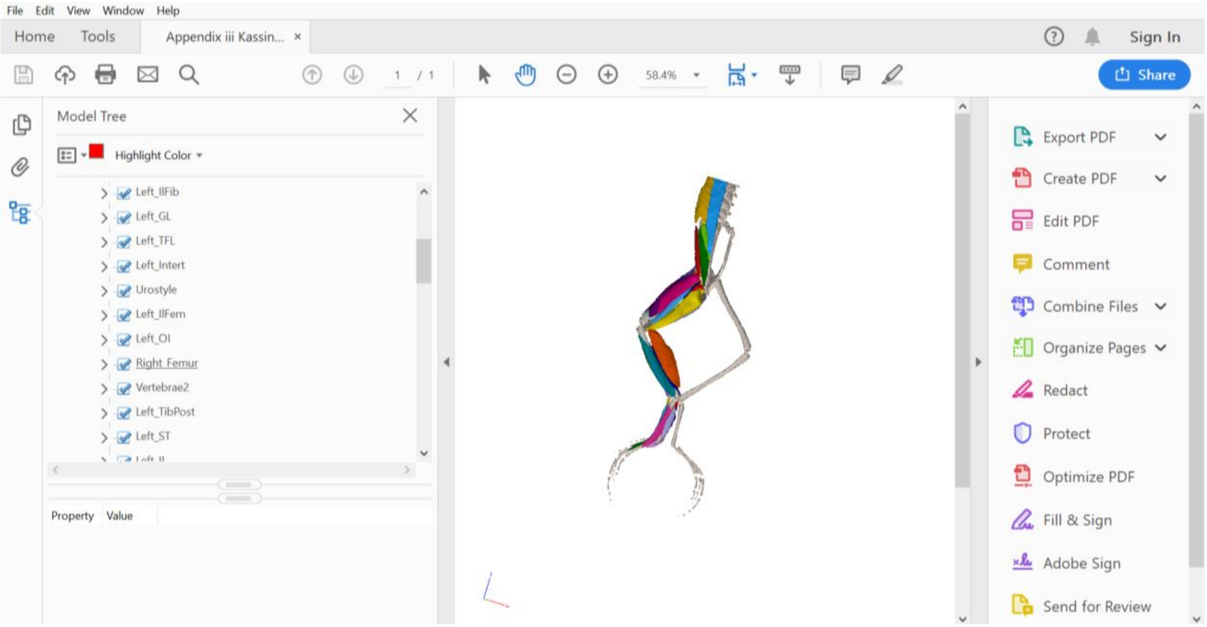

Supplement: Article S1 [file peerj-07-7003-s001.pdf]
